# Efficiency of Utilizing Bulls with High Immune Response (HIR) in Terms of Reproductive Traits of PHF Cows

**DOI:** 10.3390/ani14152144

**Published:** 2024-07-23

**Authors:** Bogumił Sobczyński, Dariusz Piwczyński, Kamil Siatka, Beata Sitkowska, Magdalena Kolenda

**Affiliations:** 1 Department of Animal Biotechnology and Genetics; Faculty of Animal Breeding and Biology, Bydgoszcz University of Science and Technology, Mazowiecka 28, 85-084 Bydgoszcz, Poland; bogumil.sobczynski@gmail.com (B.S.); darekp@pbs.edu.pl (D.P.); beatas@pbs.edu.pl (B.S.); 2 Department of Animal Breeding and Nutrition; Faculty of Animal Breeding and Biology, Bydgoszcz University of Science and Technology, Mazowiecka 28, 85-084 Bydgoszcz, Poland; kamil.siatka@pbs.edu.pl

**Keywords:** dairy cows, high immune response, reproductive performance, calving ease, breeding strategies

## Abstract

**Simple Summary:**

Simple Summary: This study examines how breeding dairy cows with high immune response (HIR) sires affects their reproductive performance. We looked at factors like calving time, how long they stay open for insemination, and the ease of calving. We found that cows related to HIR sires tended to have shorter intervals between calving, which is good for farm productivity. However, there were also more cases of twin pregnancies and difficult births among these cows. This suggests that while there are some benefits to breeding with HIR sires, there are also potential challenges. Understanding these effects can help farmers make better decisions about breeding strategies, ultimately contributing to the efficiency and success of dairy farming.

**Abstract:**

Reproductive traits in dairy cattle are crucial for herd productivity and profitability. This study investigates the influence of relatedness to high immune response (HIR) Immunity+ sires on reproductive performance indicators in Polish Holstein-Friesian cows. A total of 5094 cows were analyzed, categorized based on their relatedness to HIR Immunity+ sires, and assessed for various reproductive parameters, including age at first insemination, gestation length, days open, calving interval, and calving ease. The results showed that the level of relatedness to HIR Immunity+ sires influenced certain reproductive traits, such as service period, gestation length, and age at first and second calving. Additionally, cows related to HIR Immunity+ sires exhibited a higher frequency of twin pregnancies and more complicated births. While some benefits were observed in certain reproductive traits among cows related to HIR Immunity+ sires, such as reduced age at first insemination and shortened gestation length, the overall impact on reproductive efficiency remains inconclusive. Further studies are needed to fully elucidate the effects of using semen from HIR Immunity+ sires on reproductive performance in dairy cattle.

## 1. Introduction

The immune system, composed of cells, tissues, and various molecules [1], constitutes the body’s natural defense system, protecting against a broad spectrum of microorganisms. This system is genetically regulated and can be enhanced through genetic selection and breeding efforts [2]. This is facilitated by the relatively high heritability (0.11–0.64) of immunological response traits (AMIR—antibody-mediated immune response and CMIR—cell-mediated immune response), comparable to the heritability of production traits [1,2,3,4,5,6]. Improving traits associated with animal health and the functioning of their immune system is a commendable practice, especially considering recent consumer expectations regarding animal health and welfare, concerns about food management systems, and limited antibiotic use in animal production [3,7]. Additionally, reports indicate the significant impact of infectious diseases on pregnancy losses [8]. Thus, the statement “Unhealthy animals may not produce healthy food” [7] becomes particularly relevant in this context. Resilient animals are healthy, fertile, and easy to manage, with better longevity and consistent production, which is essential for the stability and sustainability of the dairy industry [9].

High Immune Response (HIR) technology, developed and patented by the University of Guelph, Canada, serves to identify cattle and swine characterized by an optimized immune response, thereby exhibiting higher resistance to a wide spectrum of diseases [2,3,10] and easier adaptation to heat stress conditions [4]. Individuals demonstrating a high immune response possess an innate ability to produce it at a more balanced and robust level compared to individuals with average or weak responses. In HIR dairy cattle, essentially half as many diseases occur compared to low-response cattle, and they can pass on their beneficial immune response genes to future generations, thus accumulating health benefits in the dairy herd [10]. In dairy cattle breeding, the technology gained practical significance through an exclusive license for its use obtained by the Semex Alliance Company, which selected bulls with enhanced immune response and subsequently introduced their semen to the market under the Immunity+ trademark [2]. Extensive studies conducted in Canada and the USA indicated that daughters of HIR Immunity+ bulls observed in commercial herds showed a lower incidence of mortality and metabolic and infectious diseases [11,12], such as digital dermatitis, pneumonia, and overall reduced occurrence of diseases among offsprings with high Estimated Breeding Values (EBVs) for immune response traits [1,4,13]. HIR Immunity+ cattle also exhibited a lower frequency of reproductive system disorders, including metritis or retained fetal membranes [11,12,14]. Moreover, Canadian studies [6] showed favorable correlations between overall immune response and reproductive traits, such as the number of services per conception (NS, 0.18) and service period (SP, r = 0.17). We hypothesized that reproductive indicators should improve with the cows’ immune response strengthening. A similar opinion was presented by König and May [15] based on a literature review. The aforementioned Canadian studies [6] showed low but positive genetic correlations in the range from 0.16 to 0.20 between non-return rates and Immune response (IR) traits (AMIR, CMIR) and a favorable (−0.17) genetic correlation between gestation length and AMIR. Furthermore, Heriazon et al. [16] found that phenotypically correlations between IR traits, AMIR, and CMIR with days to first service were lower than −0.11. However, the positive relationship between fertility traits and increased animal immunity did not apply to the ease of calving, which exhibited a trend opposite to the aforementioned traits (r = −0.19) [6], although another study [17] also demonstrated improvement in this trait.

Correctly managed reproduction in the herd is the cornerstone of effective management and profitable milk production [18,19,20,21,22]. The goal of breeders aiming to maximize profits should be to achieve the fastest possible reproduction after the previous calving [23]. The post-calving period consists of a sequence of consecutive events, including uterine involution, resumption of the estrous cycle, insemination, and then recognition and maintenance of pregnancy [24,25]. Nevertheless, a series of biological and physiological changes occur even before parturition during the phase of preparing to deliver offspring, milk production, and lactation [26,27]. These processes involve significant morphological, immunological, and microbiological changes, making precise modulation of the maternal immune response a prerequisite for her health and fertility [22,27,28]. Infertility or reduced fertility can result from many factors, with increasing evidence pointing to abnormal or disturbed functioning of the immune system as a cause. The immune system collaborates with all organs associated with animal reproduction, including the hypothalamus, pituitary gland, gonads, and uterus [29,30]. It should be noted that elements of this system participate in a series of functional and physiological changes related to oocyte development, maintenance of pregnancy, and even parturition [24,25]. Changes occurring in the aforementioned relationships as a result of infection or chronic immune disorders will result in fertility problems [20,23,24,25], which may manifest as prolonged anestrus, decreased conception rates, or increased rates of pregnancy loss [20,23]. Importantly, it does not matter whether the disease initiating the cascade of adverse events occurs before insemination (even several weeks before), around insemination, or at the moment of fertilization; furthermore, the condition does not necessarily have to directly affect the reproductive system [20,23], as evidenced by mastitis [7,18,21,31,32,33]. Negative impacts have also been noted in increased cases of lameness, respiratory problems, and gastrointestinal and metabolic disorders [7,23,34]. Therefore, it seems that the offspring of HIR Immunity+ bulls should be privileged compared to cows unrelated to sires characterized by enhanced immune responses. Additionally, it is worth considering that daughters of HIR Immunity+ bulls not only have a lower incidence of diseases but also exhibit better production parameters than offspring of non-Immunity+ bulls. Moreover, HIR Immunity+ bulls in these studies had higher values of production index score (by 186 points) and net merit scores (by $165) compared to non-Immunity+ sires included in the analyzed breeding value ranking [10,17]. This could potentially increase interest in the semen of these bulls among dairy breeders. According to Denholm et al. [35], the associations observed between immune-associated, health, fertility, and production traits suggest that genetic selection for cellular immune-associated traits could provide a valuable tool in improving animal health, fitness, and fertility.

The study aimed to assess the effectiveness of utilizing bulls with enhanced immune response HIR Immunity+ in improving reproductive traits, such as the insemination index, service period, calving interval, gestation length, and age at first calving of Polish Holstein-Friesian cows.

## 2. Materials and Methods

The study encompassed 5094 cows (out of which 5087 had data for the first lactation and 2665 for the second lactation) of the Polish Holstein-Friesian breed, born between 2014 and 2018, raised in 7 high-yielding farms throughout Poland. Among them, 1015 cows were offspring of bulls with enhanced immune response HIR Immunity+ (HIR), including 642 cows related to them in the first generation (group I50) and 373 cows in the second generation (I25). Individuals unrelated to HIR non-Immunity+ bulls comprised 4079 cows (group I0). The pedigrees of all examined animals included parents, grandparents, and great-grandparents from both sides. The data were obtained from the SYMLEK registration system provided by the Polish Federation of Cattle Breeders and Dairy Farmers (PFHBiPM) and concerned cows that calved between 2017 and 2019.

The study analyzed the level of the following reproductive traits (1—first reproductive period, 2—second reproductive period):

- Age of calving (AC, days) at first (AC1) and second (AC2) calving,
- Services per conception (SPC, no./conception)—number of services per conception in first parity cows (SPC1) and second parity cows (SPC2),
- Service period (SP), days—days that elapsed between the first and last insemination resulting in first (SP1) and second conception (SP2),
- Gestation length (GL), days—length of first (GL1) and second pregnancy (GL2),
- Calving to conception interval (CCI), days—number of days between calving and conception (CCI1, CCI2),
- Calving interval period (CI), days—number of days between successive calves (CI1, CI2),
- Calving ease (CE), CE1 in first parity, CE2 in second parity,

Additionally, data for each cow on the day of insemination (AI), type of pregnancy (single or twin), number of stillborn calves, milk yield, and calving ease were recorded. In terms of calving ease, cows were classified as those with unassisted delivery or requiring minor human assistance (easy) and cows requiring major human assistance (difficult) (dystocia, requiring veterinary attendance, Caesarean section).

The criteria for choosing these traits for inclusion in the study were their economic importance and the fact that they have been registered for many years as part of the official assessment of breeding value in Poland. It is worth emphasizing that the statistical analysis of the controlled traits was conducted separately for the first and second production/reproductive cycles.

Variations in reproductive traits—variables AC, GL, CCI, and CI—were tested by multifactorial analysis of variance using the least square method based on the following linear models:

Model 1 (AC1): $y_{ijkls}=\mu +a_{i}+b_{j}+c_{k}+d_{l}+{\left(cd\right)}_{kl}+e_{ijkls}$,
Model 2 (AC2): $y_{ijkls}=\mu +a_{i}+b_{j}+c_{k}+d_{l}+{\left(cd\right)}_{kl}+\beta 3X3+e_{ijkls}$,
Model 3 (GL1): $y_{ijkls}=\mu +a_{i}+b_{j}+c_{k}+d_{l}+{\left(cd\right)}_{kl}+\beta 1X1+e_{ijkls}$,
Model 4 (CCI1, CI1): $y_{ijkls}=\mu +a_{i}+b_{j}+c_{k}+d_{l}+{\left(cd\right)}_{kl}+\beta 1X1+\beta 3X3+e_{ijkls}$,
Model 5: (GL2, CCI2, CI2): $y_{ijkls}=\mu +a_{i}+b_{j}+c_{k}+d_{l}+{\left(cd\right)}_{kl}+\beta 2X2+\beta 4X4+e_{ijkls}$,

where:

*y*—the phenotype value of the trait,
µ—a general average,
*a_i_*—the fixed effect of the ith HIR (I0, I25, I50),
*b_j_*—the fixed effect of jth herd (1..7),
*c_k_*—the fixed effect of the kth year of calving (2017, 2018, 2019),
*d_l_*—the fixed effect of lth calving season (summer: V–X, winter: XI–IV),
(*cd*)_kl_—the fixed effect of the klth year of calving × season of calving,
*β*1*X*1—regression on age of first calving
*β*2*X*2—regression on age of second calving
*β*3*X*3—regression on milk yield in the first lactation
*β*4*X*4—regression on milk yield in the second lactation
*e_ijkls_*—random error.

The above-mentioned effects were estimated by applying the variance analysis (Fisher-Snedecor test) and the Scheffé test.

The analysis of count data (SPC, SP) was conducted using a generalized linear model and Poisson (SPC) and negative binomial (SP) models and log link function as follows:

SPC: $p\left\{Y|\lambda \right\}=\prod_{ijlos}\frac{\lambda_{ijmns}^{y_{ijmns}}exp{\{-\lambda }_{ijmns}\}}{y_{ijmns}!}$,

SP: $p\left\{Y|\omega ,\kappa \right\}=$ $\frac{\Gamma (\kappa +y_{ijmns})}{\Gamma \left(\kappa \right)y_{ijmns}!}$ ${\left(\frac{\kappa }{\omega +\kappa }\right)}^{\kappa }{\left(\frac{\omega }{\omega +\kappa }\right)}^{y_{ijmns}}$, where:

*λ* is a parameter of Poisson models; ω is the mean, and κ is the shape parameter of the negative binomial model; Y = {$y_{ijmns}\}$ is the vector of counting outcomes.

log (*λ*) = $\eta_{ijmn}$ + $e_{ijmns}$
log (ω) = $\eta_{ijmn}$ + $e_{ijmns}$,

where $\eta_{ijmn}$ is a function of the expected value of SPC or SP, and $e_{ijmns}$ is residual with assumption $e_{ijmns}$ ~ N(0,$\sigma_{e}^{2}$).

Model 6 (SPC1, SP1): $\eta_{ijmn}$ = $\beta_{0}+a_{i}+b_{j}+f_{m}+g_{n}+{\left(fg\right)}_{mn}+\beta 5X5$,

Model 7 (SPC2, SP2): $\eta_{ijmn}$ = $\beta_{0}+a_{i}+b_{j}+f_{m}+g_{n}+{\left(fg\right)}_{mn}+\beta 3X3+\beta 6X6,$

$\beta_{0}$—the intercept, 

*f_m_*—the fixed effect of mth insemination year (2016–2018),

*g_n_*—the fixed effect of the nth insemination season (summer: V–X, winter: XI–IV),

(*fg*)*_mn_*—the fixed effect of the mnth year of insemination × season of insemination,

*β*5*X*5—regression on the age of the first insemination in the first parity

*β*6*X*6—regression on the age of first insemination in second parity.

The rest of the symbols are the same as in the description of models 1–5.

The groups of animals, classified using the classification model, were compared based on least square means using the Scheffé test.

Subsequently, an analysis of the distribution of calving progression, pregnancy size, and the number of stillborn calves depending on the HIR gene contribution in the genotype of calving cows was conducted using the chi-square test.

The collected data was analyzed statistically using the FREQ, GLM, and GENMOD procedures of SAS software [36]. 

## 3. Results

The statistical characteristics of the reproductive traits of the cows included in the study are presented in Table 1. The statistical analysis revealed a significant and highly significant influence of HIR Immunity+ bulls on SP1, GL1, AC1, and AC2 (Table 2). Additionally, a statistically confirmed effect of AC (AI) on SPC1, SP1, GL1, GL2, and CC2 was observed.

A statistical influence of the calving or insemination year on the services per conception (SPC1, SPC2), service period (SP1, SP2), age at calving for both reproductive cycles (AC1, AC2), as well as the length of the first gestation (GL1) and first calving interval (CI1) was demonstrated (Table 2). It was also found that the calving or insemination season was a source of variability in age at calving and gestation length of primiparous heifers, as well as the services per conception, service period, and age at calving of primiparous cows. Furthermore, it was shown that the interaction between year and calving season statistically differentiated heifers and primiparous cows at their age of calving and services per conception in their second reproduction period. Additionally, it was observed that the herd was a statistically differentiating factor for all controlled traits. The statistical analysis indicated that the milk yield level statistically influenced both calving to conception interval and calving intervals.

The least-square means for the evaluated reproductive traits were computed in the subsequent statistical analysis stage. However, due to the aim of the study, only the means associated with the effect of HIR Immunity+ bull genes in the cows’ genotype were presented and discussed (Table 3). The research revealed that the number of insemination procedures (SPC) required for heifer fertilization (SPC1) varied (*p* > 0.05) from 1.47 (I25) through 1.90 (I0) to 1.60 (I50), while for primiparous cows (SPC2), it ranged from 1.72 (I25) through 1.94 (I0) to 1.94 (I50). It was demonstrated that the service period (SP1) in the group of heifers with a 25% contribution of HIR Immunity+ bull genes (I25) was statistically shorter than in the I0 and I50 groups, by 5.78 and 7.38 days, respectively. The duration of the service period in the subsequent reproductive cycle (SP2) ranged from 32.20 (I25) through 34.09 (I0) to 36.51 days (I50), but no significant differences were found. The study showed that heifers, daughters of HIR bulls (I50), were 13.68 days older at the time of their calving than peers in the I25 group (*p* ≤ 0.05) and 6.17 days older than those in the I0 group. A similar trend (*p* ≤ 0.05) regarding the order of groups was also observed in terms of the age at the second calving (AC2)—the difference between the extreme groups (I25, I50) was 52.83 days. It was found that among the compared groups of heifers (*p* ≤ 0.05) and primiparous cows (*p* > 0.05), daughters of HIR Immunity+ bulls (I50) had the shortest gestation periods (GL), respectively: first pregnancy (GL1)—276.06 days, second (GL2)—277.20 days. Simultaneously, it was observed that the first pregnancy (GL1) lasted the longest in the I0 group (277.13 days), while the second (GL2) was in the I25 group (278.17 days).

The study revealed that the length of the interval between first calving and conception (CCI1) in the I0 and I50 groups was noticeably longer than in the I25 group, although these differences were not statistically confirmed (Table 3). In turn, in terms of calving interval (CI1), exactly the opposite tendency was found. In both cases (CCI1, CI1), the differences were not statistically confirmed. Comparative analysis regarding the second calving to conception (CCI2) and calving interval (CI2) periods—limited due to the small number of cows in the I25 group only to groups I0 and I50—showed no statistically significant differences between I0 and I50. However, it is worth noting that cows in the group unrelated to HIR Immunity+ bulls (I0) exhibited a shorter calving to conception (CCI2) period but a longer calving interval (CI2) period compared to the daughters of these sires, by 3.69 and 5.51 days, respectively.

Table 4 presents the results concerning the course of calving and the number of born calves and stillborn calves in the studied population. Statistically significant differences were observed in the case of primiparous heifers regarding calving ease and the number of born calves. With the increasing proportion of HIR Immunity+ bull genes, a decrease in the proportion of easy calving in their total number was noted, and twin births were more frequently observed. The frequency of multiple pregnancies in this group of animals was over three times higher than in all calving heifers (3.95% vs. 1.28%). There were no significant differences in the number of stillborn calves. The presence of HIR genes was indicated as an important factor for the frequency of observed multiple pregnancies in the second reproductive season. They occurred most frequently in I50 cows, where they were noted almost 1.8 times more often than in all second-calving animals (9.09% vs. 5.19%). The level of relatedness did not significantly differentiate the results related to ease of parturition and stillbirths.

## 4. Discussion

### 4.1. General Characteristics of the Studied Population

Based on the reproductive traits presented in Table 1, it was shown that the heifers evaluated in this study entered reproduction relatively late, as the first insemination occurred on average at 476.35 days of age (15.9 months). The age at first calving, amounting to 780.49 days (26 months), fell within the recommended limits for the Holstein-Friesian breed [37,38,39,40] and was better than in the case of cows evaluated for breeding value in Poland in 2022, which averaged 798 days. For Holstein-Friesian cattle of the black-and-white variety, AFC was 792 days, and for the red-and-white variety, 814 days [37]. Similarly, in the study by Pytlewski et al. [41], the age at first calving was longer than in our study, at 791.60 days (26 months).

The number of services required to achieve both the first (1.65) and second pregnancies (1.94) can be considered satisfactory. According to Mordak [42], this result should oscillate around 1.5 in very well-managed herds, while a value close to 2 is considered acceptable. Borkowska et al. [40] indicate that the expected value for this indicator falls within the range of 1.6 to 1.8.

The calving to conception intervals in the first and second lactations, at 84.32 days and 80.62 days, respectively, are very good results compared to those reported for animals evaluated by PFHBiPM (137 days) [37]. At the same time, they were shorter than those recommended in the literature (85–100 days) [37,42]. 

The calving intervals periods obtained in the first and second reproductive cycles (397.57 and 400.58 days) were within the range recommended for Polish Holstein-Friesian cattle [38,39,40,43], however, were significantly shorter than the average inter-calving periods observed in Polish Holstein-Friesian cattle of both color varieties in 2022 (422 days for black-and-white cattle and 420 days for red-and-white cattle), as well as for the entire population evaluated for breeding value (422 days) [37]. 

It should be emphasized that the animals included in this study exhibited significantly higher average lactation yields (11,076.64 and 11,987.35 kg) compared to cows in the national assessment conducted by PFHBiPM (HO 9315 kg, RW 8276 kg, and a total of 9037 kg in the evaluated population) [37]. Therefore, it can be concluded that the animals assessed in this study generally showed very good reproductive indicators compared to cattle evaluated for breeding value in the country, especially when compared to fertility indicators obtained from the active population in 2020 [44], which is the most recent data available for cows considered in this analysis. Cows evaluated for value in Poland until the end of 2020 had an AC1 of 804 days, CI of 430 days, CCI of 146 days, and GL of 279 days.

Fertility indices obtained in our study also compare favorably with data from other parts of the world. For example, Muller et al. [45], analyzing the fertility of Holstein cattle maintained in South Africa, reported that the calving to conception interval (CCI) averaged 133.9 days. The calving to first service interval was 77 ± 30 days, with only 64% of first services occurring within 80 days postpartum and an average of 2.55 insemination procedures required to achieve pregnancy. Recent reports from Iran [19] indicate that in six local Holstein cattle herds, reproductive indices for cows ranged from CCI 123 to 154 days, CI 398 to 445 days, and services SPC 1.9 to 2.5. Results for heifers were slightly more favorable than those obtained in our study, with age at first service ranging from 459 to 481 days, AC1 749 to 786 days, and SPC 1.34 to 1.47. 

### 4.2. The Influence of Selected Factors on the Level of Analyzed Reproduction-Related Traits

The impact of individual main factors (HIR, Age at insemination or calving, Year and season of calving, Herd, and Milk Yield) and the included interactions between Year × Season of calving on the estimated reproductive parameters varied depending on the analyzed reproductive period, as detailed in Table 2. Our research results are only partially consistent with a series of reports from other authors.

The influence of the herd on reproductive efficiency was confirmed by Zahedi et al. [19]. Iranian reports indicate that generally, two- and three-way interactive effects of herd, year, and season of calving were significant on reproductive parameters (AC1, DFS, calving to first service, CI, AFSage of first service, DO, SPC-services per conception, and CR-conception rate %) of cows and heifers (*p* < 0.01). The impact on the reproductive efficiency of Holstein-Friesian cattle measured by the effectiveness of the first insemination due to the interaction of subsequent lactations, herd (size and performance), insemination season as a main factor, and in interaction with daily performance, was also confirmed by Siatka et al. [18,46]. Pytlewski et al. [41], analyzing the reproduction results of Polish Holstein-Friesian cows that exceeded the threshold of life performance of 100,000 kg of milk, identified AC1, cow age (lactation), and season as differentiating factors for reproductive indices. Season of calving significantly influenced the Insemination Index and GL. The number of lactations and season of calving as significantly differentiating GL was also indicated in American reports [47].

In the studies conducted by Boujenane and Draga [48], all reproductive traits (age at first, gestation length, days from calving to first insemination, days open, days from first insemination to conception, calving interval, number of inseminations per conception, and success of conception at first insemination) were significantly (*p* < 0.001) influenced by herd and year.

### 4.3. Services per Conception

It is widely acknowledged that the early lactation period is the time of greatest risk for the development of various diseases in cattle, both clinical and subclinical. The literature indicates that in the first few weeks after calving, health problems affect over 50% of animals [26]. According to Pytlewski et al. [41], a way to improve reproductive outcomes and simultaneously prolong animal longevity could be selection for improved resistance in cows, supported by reports on correlations between clinical mastitis and other economically important traits by other researchers. The negative impact of subclinical mastitis on SPC has been demonstrated, among others, by Nuraddis et al. [21]. Cows identified as healthy required about 1 less insemination for pregnancy compared to cows with subclinical mastitis (1.59 vs. 2.51). This was also reflected in the first insemination success rate, which was significantly higher in healthy cows (56.1% vs. 32.6%). Positive correlations between antibody-mediated immune responses (AMIR) and reproductive traits such as SPC (r = 0.18) and SP (r = 0.18) were noted in heifers by Thompson Crispi [5]. Considering that the optimum value for SPC is considered to range between 1.6 and 1.8 [18], the results obtained in this study for SPC should be regarded as good, albeit not allowing the conclusion that increased immunological response is a significantly reducing factor in the number of inseminations needed for pregnancy. Interestingly, the best SPC indices (*p* > 0.05) were obtained in heifers and cows from the I25 group, contrary to what might be assumed based on the above reports of animals from the I50 group (Table 3).

### 4.4. Service Period

The results obtained in our study suggest that while the presence of HIR Immunity+ sires in the pedigree of cows in the second reproductive season does not affect SP, for heifers, being related to them by 25% proved beneficial (Table 3). It is worth noting that cows from the I50 group exhibited the longest SP1. This result is somewhat reflected in the findings presented by Scott et al. [35], who observed negative phenotypic correlations between the percent CD4+ lymphocytes and the time between the first and second service (r = −0.14), as well as a positive relationship between percent monocytes and CI (0.10). Additionally, they noted a negative association between percent eosinophils and reproductive episodes (r = −0.25).

When analyzing SP, one must consider reports such as those by Gegenfurtner et al. [49], which indicate that a significant proportion of embryonic loss in dairy cows occurs quite early after conception. According to the cited authors, the loss of embryos prior to Day 7 is as high as ∼50%. Generally, there are four crucial periods for pregnancy loss during the first trimester of gestation in lactating dairy cows. The first occurs during the first week after breeding due to fertilization failure or death of the early embryo (20–50%), the second from days 8 to 27 with losses averaging ~30% but ranging from 25% to 41%, the third from days 28 to 60, with losses of ~12%, and the last during the third month of pregnancy (~2%).

### 4.5. Age at Calving

It is commonly acknowledged that heifers are inseminated or serviced when they reach the so-called breeding maturity, which is determined primarily by the development of their organs and body weight rather than age. In the case of Polish Holstein-Friesian heifers, the first insemination is typically performed at 14–16 months [41]. This means that AC1 should occur at 23–25 months. Our results indicate that the optimal AC was exceeded regardless of the presence of HIR Immunity+ sires. Heifers that gave birth to offspring the fastest were those from the I25 group (Table 3). Therefore, it can be assumed that these animals exhibited the best body weight gains during the rearing period. Confirmation of these assumptions can be found in the results presented by Grala et al. [24]. Heifers with HiAv-IR (high and average IR) had a more significant average daily weight gain from 13 to 52 weeks of age (661 g vs. 619 g) and tended to be younger at puberty (371 days vs. 385 days) than low-IR heifers. Other research groups have also investigated the relationship between daily weight gain and the functioning of the immune system. König and May [15] indicated that High AMIR calves had a higher average daily gain compared with low AMIR calves (0.72 ± 0.02 vs. 0.66 ± 0.06 kg/d; *p* = 0.009). It is also worth considering reports concerning Irish dairy cattle females in their humoral immune response to BoHV-1 (Bovine herpesvirus-1) and the genetic association of humoral immune response to BoHV-1 with milk and fertility performance traits [50] that suggest that breeding for resistance to BoHV-1 infection may indirectly improve fertility performance.

The obtained AC2 is a consequence of a series of events occurring after the first calving and the breeder’s decision to reintroduce the animals for reproduction. In this case, it should be noted that they reflect the results concerning AC1. The youngest in their second calving group were cows from the I25 group; however, unlike AC1, the differences between all groups were statistically confirmed (Table 3). The reasons for these differences between groups can be attributed to the accumulation within each of them of mostly small differences (*p* > 0.05) observed in parameters such as AC1, CCI, and GL.

### 4.6. Gestation Length

Gestation length (GL) is a moderately heritable trait in cattle with economic and management implications. GL measures the period of prenatal development from conception to birth [51]. The physiological GL in cattle typically ranges from 270 to 290 days, with a mean of 280 days, and it is influenced by various factors such as the age of the cows, breed, sex of the fetus, number of fetuses, freedom of movement provided for the pregnant female, and environmental conditions including calving season and nutrition [41,51,52]. Younger cows tend to have shorter gestations, while longer gestations are observed when calving occurs during cooler seasons (autumn and winter), which applies to both heifers and older cows [51]. All cows involved in this study exhibited GL within the physiological range. Small (±1 day) but statistically confirmed differences were observed only for Parity 1, where the shortest gestations were observed in the I50 group (Table 3). This information is valuable for accurate calving date prediction, and consequently, GL estimation is crucial for effective herd management [52].

Considering the findings of Nogalski and Piwczyński [53], the fact that GL ranged from 276.06 to 278.12 days indicates that it was within the optimal range for Polish Holstein-Friesian cows. Those authors reported that both prolonged and shortened GL contributed to a significant increase in the number of stillbirths and assisted calving. Other reports also suggest that intermediate GL is optimal for calving ease and stillbirth rate [51]. However, the results of our study do not confirm the findings of Thompson-Crispi et al. [6], which indicated a positive correlation between HIR Immunity+ and GL (0.17).

### 4.7. Calving to Conception Intervals

Successful reproduction requires the birth of a single, live, and viable calf within 12 to 14 months of the previous calving, depending on the production system. This necessitates prompt reconception within a limited and defined period after the previous parturition, often about 100 days, successful maternal recognition of pregnancy, and normal growth and development of the embryo and fetus [23]. On the other hand, the preferred length of the postpartum resting period, the period between calving and the first artificial insemination or mating service, in a given reproduction cycle should be at least 6 weeks. This period is connected with the time required for cows to prepare for the subsequent pregnancy [41]. Siatka et al. [18] demonstrated that cows inseminated <60 days in milk exhibited the highest SPC (2.41), which decreased with lactation progression. This finding is consistent with other reports indicating the optimal period between 70 and 90 days post-calving as most conducive to reconception and first insemination, although in the case of cows with an average yield >12,000 kg, a later period can be considered [41,48,54]. Considering the above, the results obtained in our study (Table 3), which are close to the upper limit of the expected values, should be considered correct for both Parity 1 and Parity 2. In both analyzed periods, although not statistically significant, there was a tendency for slightly later reconception in cows related to HIR bulls. These results deviate from reports in the literature [6] indicating beneficial correlations between high immune response with a 56-day non-return rate (0.16), numbers of services to conception (0.20), first service to conception (0.18), suggesting that animals related to HIR bulls should have better reproductive performance [10]. 

The influence of immune response (IR) on reproductive processes has also been demonstrated in a management system based on seasonal reproduction in the herd [24]. It was found that Low-CMIR cows of NEG FertBV (a negative fertility breeding value) had a >40-day longer calving to first ovulation interval during their first lactation compared with HiAv-CMIR (NEG FertBV cows. Additionally, Low-CMIR ows also had decreased pregnancy rates at both 3 weeks (25% vs. 42%) and 6 weeks (33% vs. 54%) into the seasonal breeding period during their first lactation, compared with HiAv-CMIR cows. Based on the findings mentioned above, it can be concluded that CMIR ranking affects the ability to achieve postpartum estrus and, thus, the possibility of reconception.

### 4.8. Calving Interval

Previous business calculations [43] suggest that for cows with a 305-day milk yield ranging up to 9000 kg, optimal economic outcomes occur when cows calve within a period of 340 to 370 days postpartum (pp). Similarly, cows producing up to 10,000 kg are more profitable, with calving intervals falling between 371 and 400 days pp. Moreover, cows yielding between 10,000 kg and 11,000 kg of milk demonstrate clear financial benefits with calving intervals spanning 400 to 430 days. The results obtained in our study regarding CI (Table 3) should be considered favorable from the perspective of milk production economics. However, in both reproductive periods, statistically confirmed differences were not observed between cows with varying degrees of relatedness to HIR Immunity+ sires. However, both in the case of Parity 1 and Parity 2, there was a tendency towards approximately 4–6 days shorter CI in the I50 groups compared to the I0 groups. Considering that the length of CI is the result of both gestation length (GL) and calving to calving interval (CCI), as well as the results obtained for these indicators in this study, this result can be considered predictable and consistent with the authors’ expectations. The influence of IR on CI length was confirmed in studies involving Brahman cattle [55]. Cows classified as High CMIR (high cell-mediated immune response individuals) were likelier to remain in the herd longer, produce a calf each year, and have shorter calving intervals (about 25 days) than their Low CMIR herd mates.

### 4.9. Calving Ease

The results presented in Table 4 indicate that the degree of relatedness of cows to HIR Immunity+ sires influenced calving ease (CE) in both Parity 1 and Parity 2. In both cases, cows without Immunity+ sires in their pedigrees exhibited higher CE than those related to them. This finding contradicts the findings of Mallard et al. [10,17], which suggest that daughters of Immunity+ sires have beneficial correlations with calving ease compared to those of non-Immunity+ sires. They suggest that producers can selectively breed for improved immune response using HIR technology without necessarily reducing genetic gain in other important traits, such as pregnancy rate and calving ease [17]. On the other hand, Thompson-Crispi et al. [6] demonstrated a negative correlation between HIR Immunity+ and CE (r = −0.19).

### 4.10. Number of Born Calves

Multiple births in cattle are a naturally occurring reproductive phenomenon. For dairy cattle, twinning is considered costly to producers due to its potentially detrimental impact on both the cow and the calf, including risks such as abortion, dystocia, stillbirth, retained placenta, metabolic disorders, displaced abomasum, ketosis, and eventual culling from the herd [56,57,58,59]. The number of calves born per cow was determined in this study to be dependent on the degree of relatedness of cows to HIR Immunity+ sires (see Table 4). In both Parity 1 and 2, the highest proportion of twin pregnancies was observed in cows with I50 ancestry. The frequency of twin pregnancies in this study exceeded that reported by American researchers [47], who found that for Holstein and HF × Jersey cattle with a 305-day lactation yield of 11,990 kg, twinning occurred at a rate of 1.9%, with a stillbirth frequency of 0.9%. However, our results fell within the typical range for dairy cattle (3 to 5%) and were similar to those obtained for high-yielding cows, where twinning reached 6–7% in second parity cows [57]. Only I50 cows in Parity 2 exceeded this rate. López-Gatius et al. [60] indicate that the risk of twin pregnancy and associated disadvantages is much more common in older cows. For example, the risk of pregnancy loss during the first trimester of gestation for cows carrying twins may be three to seven times higher than for cows carrying singletons. A higher frequency of twin pregnancies in multiparous cows was also indicated in a review of the economic consequences of twin pregnancies in dairy cattle by Cabrerra and Fricke [59]. Breeders using semen from HIR Immunity+ sires should also consider the findings of Lett and Kirkpatrick [56], who point out that while twinning depends on many factors (herd, year, season, parity), it is also an inheritable and repeatable trait.

### 4.11. Stillborn Calves

Stillbirths are an economically significant trait in dairy farming. It is estimated that a single case of stillbirth generates losses of approximately US$ 938 (ranging from $US 767 to $US 1189). Stillbirths not only entail financial losses associated with the loss of the calf but are also positively correlated with other reproductive disorders [52,61], such as the risk of developing metritis and retained placenta, increased SPC among primiparous cows, or increased risk of culling from the herd due to low reproductive performance. Understanding the consequences of stillbirths facilitates decision-making for breeders [61]. In this study, the stillbirth rate was very low, not exceeding 1%, regardless of parity or the degree of relatedness to HIR Immunity+ sires (see Table 4). The degree of relatedness to HIR Immunity+ sires did not prove to be a differentiating factor in the frequency of stillbirths.

## 5. Conclusions

The level of relatedness of cows to HIR Immunity+ sires influenced AC, including a reduction in AC1, which can be considered advantageous. The level of relatedness of cows to HIR Immunity+ sires also affected the length of SP and GL in the first reproductive period (Parity 1). However, due to the lack of influence on other analyzed parameters, significant from an economic point of view, such as SPC, CCI, and CI, as well as the more frequent occurrence of twin pregnancies observed in cows related to HIR Immunity+ sires, it is difficult to confirm the benefits of using semen from HIR Immunity+ sires for reproductive efficiency based on these results, as indicated by other authors. The level of relatedness of cows to HIR Immunity+ sires influenced CE generated a greater number of more difficult births in the first reproductive period (Parity 1) and second (Parity 2).

## Figures and Tables

**Table 1:** The statistical characteristic of reproduction traits and milk yield.

Trait	*n*	Mean	Q1	Median	Q3	SD	CV	Kurtosis	Skewness
	**First reproduction cycle/lactation**								
AI (days)	4944	476.35	449	475	496	43.72	9.18	4.72	1.09
SPC1	4944	1.65	1	1	2	0.99	59.9	4.27	1.88
SP1 (days)	4432	24.95	1	1	36	44.84	179.69	10.42	2.82
AC1 (days)	5087	780.49	739	766	808	66.3	8.5	3.65	1.46
GL1	4562	276.66	273	276	280	5.45	1.97	1.67	0.50
CCI1 (days)	1559	84.32	57	71	97	41.71	49.47	5.09	2.03
CI1 (days)	2658	397.57	347	378	430	69.52	17.49	4.22	1.65
MY1 (kg)	5087	11,076.46	9267.3	11,356.9	13,423.6	4302.43	38.84	0.9	−0.33
	**Second reproduction cycle/lactation**								
AI (days)	2606	857.04	804	846	898	75.13	8.77	1.5	0.87
SPC2	2606	1.94	1	1	2	1.29	66.77	6.35	2.03
SP2 (days)	2201	38	1	1	59	58.66	154.34	8.18	2.42
AC2 (days)	2665	1172.9	1106	1158	1226	95.6	8.15	1.55	0.93
GL2	2355	277.69	274	278	281	5.76	2.07	1.44	0.17
CCI2 (days)	602	80.62	59	70	93	35.56	44.11	7.71	2.23
CI2 (days)	1017	400.58	348	383	432	69.31	17.3	4.19	1.65
MY2 (kg)	2665	11,987.35	9639.8	12,415.2	14,689.5	4313.61	35.98	0.44	−0.42

AI—age of insemination, SPC—services per conception, SP—service period, AC—age of calving, GL—gestation length, CCI—calving to conception interval, CI—calving interval period, MY—milk yield, 1—first reproduction cycle/lactation, 2—reproduction cycle/lactation. Q1—lower quartile, Q3—upper quartile, SD—standard deviation, CV—coefficient of variation (%).

**Table 2:** The significance of the impact of main factors and second-degree interactions on the investigated traits (Probability).

Trait	HIR	Calving (Insemination) Age	Year (Y)	Season (S)	Y × S	Herd	Milk Yield
First							
SPC1	0.0823	<0.0001 ^i^	<0.0001 ^i^	0.2783 ^i^	0.9999 ^i^	<0.0001	
SP1	0.0068	<0.0001 ^i^	<0.0001 ^i^	0.5602 ^i^	0.3638 ^i^	<0.0001	
AC1	0.0115		<0.0001 ^c^	0.0380 ^c^	0.0001 ^c^	<0.0001	
GL1	0.0001	<0.0001 ^c^	0.0435 ^c^	<0.0001 ^c^	0.9054 ^c^	<0.0001	
CCI1	0.2401	0.7601 ^c^	0.0867 ^c^	0.3446 ^c^	0.6614 ^c^	<0.0001	<0.0001
CI1	0.1707	0.5464 ^c^	0.0004 ^c^	0.2868 ^c^	0.1868 ^c^	<0.0001	<0.0001
Second							
SPC2	0.4118	0.3332 ^i^	<0.0001 ^i^	0.0017	0.0488	<0.0001	0.4841
SP2	0.7558	0.6266 ^i^	<0.0001 ^i^	0.0069	0.0041	<0.0001	0.6525
AC2	<0.0001		<0.0001 ^c^	0.0416 ^c^	<0.0001 ^c^	<0.0001	0.2538
GL2	0.0705	0.0104 ^c^	0.4863 ^c^	0.3130 ^c^	0.3682 ^c^	<0.0001	0.1816
CCI2	0.2057	0.0471 ^c^	0.7528 ^c^	0.9689 ^c^	0.9659 ^c^	<0.0001	<0.0001
CI2	0.2269	0.1547 ^c^	0.1180 ^c^	0.3985 ^c^	0.2783 ^c^	<0.0001	<0.0001

SPC—services per conception, SP—service period, AC—age of calving, GL—gestation length, CCI—calving to conception interval, CI—calving interval period, 1—first reproduction cycle/lactation, 2—reproduction cycle/lactation, ^i^—year and season of insemination, ^c^—year and season of calving.

**Table 3:** Average values of reproductive traits with respect to the HIR.

Traits	Measure	Parity 1			Parity 2		
		I0	I25	I50	I0	I25	I50
	*n*	3958	371	615	2146	57	403
SPC	LSM	1.57	1.47	1.60	1.90	1.72	1.94
	SE	0.02	0.04	0.04	0.03	0.15	0.07
	*n*	3535	332	565	1799	41	361
SP	LSM	21.46 A	15.68 Aa	23.06 a	34.09	32.20	36.51
	SE	0.87	1.47	1.91	1.66	8.28	3.52
	*n*	4076	373	638	2189	59	417
AC	LSM	786.57	779.06 a	792.74 a	1178.51 Aa	1145.21 Ba	1198.04 AB
	SE	1.30	3.42	3.01	2.55	12.11	4,99
	*n*	3645	343	574	1933	50	372
GL	LSM	277.13 A	277.02	276.06 A	277.99	278.17	277.20
	SE	0.11	0.30	0.26	0.15	0.81	0.33
	*n*	1284	40	235	489		113
CCI	LSM	82.13	91.28	83.04	86.08		90.77
	SE	3.68	6.12	4.24	4.01		4.91
	*n*	2186	59	413	814		203
CI	LSM	393.37	400.14	389.02	405.49		399.98
	SE	5.20	7.79	5.67	5.88		6.95

SPC—services per conception, SP—service period, AC—age of calving, GL—gestation length, CCI—calving to conception interval, CI—calving interval period, LSM—least means square, SE—standard error, AA (aa)—Values within the reproductive cycle that are significantly different within a variable are marked with the same letters *p* ≤ 0.01 (*p* ≤ 0.05), HIR Immunity+ I0—non-Immunity+, I25—first generation Immunity+, I50—second generation Immunity+, AA (aa)—means marked with the same uppercase (lowercase) letters differ at *p* ≤ 0.01 (*p* ≤ 0.05).

**Table 4:** Distribution of ease of calving and number of live-born and stillborn calves depend on parity 1 and parity 2.

Trait	Level	*n*/%	I0	I25	I50	I0 + I25 + I50	*p*	I0	I25	I50	I0 + I25 + I50	*p*
Calving easy	Easy	*n*	3561	327	531	4419	0.006	2111	55	395	2561	0.239
		%	87.99	87.67	83.49	87.40		96.88	94.83	95.41	96.61	
	Difficult	*n*	486	46	105	637		68	3	19	90	
		%	12.01	12.33	16.51	12.60		3.12	5.17	4.59	3.39	
No of born calves	1	*n*	3701	352	560	4613	<0.0001	2003	49	360	2412	0.0007
		%	99.14	98.60	96.05	98.72		95.52	96.08	90.91	94.81	
	2	*n*	32	5	23	60		94	2	36	132	
		%	0.86	1.40	3.95	1.28		4.48	3.92	9.09	5.19	
	Total		3733	357	583	4673		2097	51	396	2544	
Stillborn calves	No	*n*	3725	357	583	4665	0.3646	2089	51	393	2533	0.5168
		%	99.79	100.0	100.0			99.62	100	99.24		
	Yes	*n*	8	0	0	8		8	0	3	11	
		%	0.21	0.00	0.00			0.38	0.00	0.76		
	Total		3733	357	583	4673		2097	51	396	2544	

HIR Immunity+ I0—non-Immunity+, I25—second generation Immunity+, I50—first generation Immunity+.

## Data Availability

Data is contained within the article.
